# Novel pathogenic variant in *MED12* causing non-syndromic dilated cardiomyopathy

**DOI:** 10.1186/s12920-023-01780-9

**Published:** 2023-12-21

**Authors:** Serwa Ghasemi, Mohammad Mahdavi, Majid Maleki, Iman Salahshourifar, Samira Kalayinia

**Affiliations:** 1 Department of Biology, Science and Research Branch, Islamic Azad University, Tehran, Iran; 2 Rajaie Cardiovascular Medical and Research Center, Iran University of Medical Sciences, Tehran, Iran; 3 Cardiogenetic Research Center, Rajaie Cardiovascular Medical and Research Center, Iran University of Medical Sciences, Tehran, Iran

**Keywords:** Whole-exome sequencing, Dilated cardiomyopathy, *MED12*, Protein-protein docking, Variant

## Abstract

**Background:**

Dilated cardiomyopathy (DCM) is a major cause of sudden cardiac death and heart failure. Up to 50% of all DCM cases have a genetic background, with variants in over 250 genes reported in association with DCM. Whole-exome sequencing (WES) is a powerful tool to identify variants underlying genetic cardiomyopathies. Via WES, we sought to identify DCM causes in a family with 2 affected patients.

**Methods:**

WES was performed on the affected members of an Iranian family to identify the genetic etiology of DCM. The candidate variant was segregated via polymerase chain reaction and Sanger sequencing. Computational modeling and protein-protein docking were performed to survey the impact of the variant on the structure and function of the protein.

**Results:**

A novel single-nucleotide substitution (G > A) in exon 9 of *MED12*, c.1249G > A: p.Val417Ile, NM_005120.3, was identified. The c.1249G > A variant was validated in the family. Bioinformatic analysis and computational modeling confirmed that c.1249G > A was the pathogenic variant responsible for the DCM phenotype.

**Conclusion:**

We detected a novel DCM-causing variant in *MED12* using WES. The variant in *MED12* may decrease binding to cyclin-dependent kinase 8 (CDK8), affect its activation, and cause alterations in calcium-handling gene expression in the heart, leading to DCM.

## Introduction

Dilated cardiomyopathy (DCM) is the most common cause of heart failure and transplantation, with an estimated prevalence of 40 cases per 100,000 individuals, accounting for around 60% of childhood cardiomyopathies [1]. Left ventricular or biventricular dilation alongside impaired contractility with left ventricular ejection fractions below 40% is defined as a characteristic of DCM [2, 3]. Concerning DCM etiology, in addition to the involvement of environmental factors, DCM-causing genes such as *LMNA, MYH7, TNNT2, TTN, RBM20*, and *BAG3* have been identified [3, 4]. The *MED12* gene maps to Xq13.1 and has 1 transcript (NM_005120.3), 45 exons, and 6925 bp nucleotides. The gene encodes the mediator of the RNA polymerase II transcription subunit 12 protein, a member of the mediator complex (MED), with a length of 2178 amino acids [5]. This protein acts as a gene-expression regulator in all eukaryotes and plays a role in developmental signaling pathways [6]. Baskin et al. [5] demonstrated that MED12 directly stimulated the transcriptional activity of myocyte enhancer factor 2 and that it was required for the maintenance of cardiac function by regulating calcium-handling genes through interactions with myocyte enhancer factor 2. Another study in 2012 showed MED12 involvement during the differentiation of vascular endothelial cells [7]. Rocha et al. [8] studied mouse embryonic stem cells and reported serious abnormalities during the heart development of *MED12* variants, resulting in cardiac dysfunction. In the present study, with the aid of whole-exome sequencing (WES), we discovered a novel pathogenic *MED12* missense variant, c.1249G > A: p.Val417Ile, which may cause DCM. To our knowledge, the current study is the first report of this variant of the *MED12* gene in patients with DCM worldwide.

## Materials and methods

### Study subject and ethics statement

In this study, an Iranian family with 2 DCM-affected children was referred to the Cardiogenetics Research Center, Rajaie Cardiovascular Medical and Research Center, Tehran, Iran, for genetic evaluation. The father and the mother, 29 and 24 years of age, respectively, were healthy according to clinical tests. One spontaneous abortion was reported in the family. The first DCM-affected offspring of the family was a candidate for heart transplantation but died at 6 years old. The proband was a 3-year-old boy without syndromic features such as intellectual disability, metabolic disorders, and skeletal muscle disease. He was diagnosed with DCM during the initial cardiovascular workup, including magnetic resonance imaging and electrocardiography.

### WES and segregation analysis

Genomic DNA samples were extracted from peripheral blood using a DNSol Midi Kit (Roche: Product No. 50,072,012) from all the family members. WES was conducted on the proband at Macrogen (Seoul, South Korea). Exome capture and exom sequencing were performed through SureSelect XT Library Prep Kit and an Illumina HiSeq 4000 Platform using 100-bp paired-end reads, respectively. For sample sequencing, read quality value > 20 and the sequencing depth (mean exome coverage (of 100X were considered. Raw reads were collected for quality control using FastQC. Alignment to the human reference genome (GRCh37/hg19) has been performed after removing low-quality reads using the Burrows–Wheeler Aligner (BWA-MEM v.07.17) [9].

After that, duplicated reads were excluded, and the Genome Analysis Toolkit (GATK, v.4.1.4.1) [10] was used to call insertion-deletions (InDels) and single-nucleotide polymorphisms (SNPs). Variant quality score recalibration (VQSR) was performed on the GATK HaplotypeCaller generated file. All variants were annotated by ANNOVAR [11]. For filtering and prioritization, intronic and synonymous variants were excluded. Then, variants with a minor allele frequency of 0.005 according to the Exome Aggregation Consortium (ExAC), the 1000 Genomes Project, the Genome Aggregation Database (gnomAD), and the ESP6500 data set were excluded. All the called variants were confirmed by inspection using Combined Annotation-Dependent Depletion (CADD) (cadd.gs.washington.edu), Sorting Intolerant From Tolerant (SIFT) (https://sift.bii.a-star.edu.sg), Polymorphism Phenotyping v2 (PolyPhen-2) (genetics.bwh.harvard.edu/pph2), MutationTaster (www.mutationtaster.org), and Protein Variation Effect Analyzer (PROVEAN) (provean.jcvi.org). Finally, variant interpretation was done using the 2015 guidelines of the American College of Medical Genetics and Genomics (ACMG) [12]. To assess the conservation of the wild-type amino acid in MED12, we conducted a multiple sequence alignment using the CLUSTALW web server (https://www.genome.jp/tools-bin/clustalw).

The bioinformatic analysis was followed by segregation analysis among all the family members, and the results were validated by polymerase chain reaction (PCR) and direct Sanger sequencing. Primer pairs were designed using Gene Runner v.6.0 with a forward sequence of 5′-TTGGTTTGGCACTACTCACTG-3′ and a reverse sequence of 5′-GGTTACAAAGGGAGTCAAGAGA-3′. The PCR mix consisted of 1.5 mmol/L of MgCl2, 10 pmol/L of primers, 1 U of Taq DNA polymerase (Amplicon, UK), 200 mmol/L of dNTP, and 100-ng genomic DNA templates. The PCR test was carried out at 95 °C for 5 min and 35 cycles (of 30 s at 95 °C, 30 s at 60 °C, and 30 s at 72 °C), followed by 72 °C for 10 min. The sequences of the PCR products were determined using the ABI Sequencer 3500XL PE (Applied Biosystems) and were analyzed using FinchTV 1.4.0.

### *In silico* study

#### MED12 and the mediator complex

MED12 is the subunit of the mediator kinase module in a mediator complex [13]. The mediator complex is an essential agent for all RNA polymerase II-mediated transcriptions [14]. Other subunits of the kinase module include cyclin-dependent kinase 8 (CDK8), cyclin C, and MED13, with MED12 activating CDK8 [15]. CDK8 phosphorylates a broad variety of substrates concerned with DNA repair, transcription, and metabolic processes [16]. Due to the old model of CDK8 kinase activity stimulation, it seems that the N-terminal fragment of MED12 ties to a surface groove on cyclin C, which binds to CDK8 via a negatively charged surface groove [17]. Unlike the activation mechanism of cyclin-dependent kinases, T-loop phosphorylation is not required for full CDK8 activity. Indeed, CDK8 T-loop phosphorylation is replaced by the binding of MED12 to CDK8, resulting in CDK8 activation [18, 19].

The latest findings concerning CDK8 activation by MED12 are related to the studies of Felix Klatt et al., [15], who showed that the N-terminal fragment of MED12 wrapped around the CDK8 molecule to locate an “activation helix” in the nearness of CDK8 T-loop. The authors also reported that, for CDK8 activation, the exact placement of the helix, not MED12 binding alone, was crucial. Moreover, their findings demonstrated that MED12-CDK8 binding blocked the binding of type II kinase inhibitors to the active site of CDK8.

#### Protein structure modeling with AlphaFold2

Since it was impossible to model a huge MED12 protein with AlphaFold2 (https://colab.research.google.com/github/sokrypton/ColabFold/blob/main/AlphaFold2.ipynb) methods [20], the InterPro (https://www.ebi.ac.uk/interpro/) web server [21] was first employed to identify and select the desired domains of the MED12 protein according to the study of Klatt, et al. [15]. Then, the FASTA file of the protein was obtained from the UniProtKB database (https://www.uniprot.org/). With the aid of the MMseqs2 server, AlphaFold2 was employed to predict the 3D structures of the native MED12 protein and its Val417Ile variant.

After that, the VAST (https://structure.ncbi.nlm.nih.gov/Structure/VAST/vast.shtml) web server [22] and the AlphaFold2 structure for the native human MED12 were utilized to predict the possible function of the domain in which the modified amino acid was located. For the evaluation of the quality of the predicted MED12 (the normal and the Val417Ile variant) structure, the Ramachandran Plot was generated employing PROCHECK v.3.5 Server (https://www.ebi.ac.uk/thornton-srv/software/PROCHECK/) [23].

#### Protein-protein docking

Several bioinformatic and web-based tools were used to perform computer-assisted molecular docking studies. First, the 3D structures of MED12 (the normal and the Val417Ile variant) were predicted with AlphaFold2. Next, the 3D structure of CDK8 was downloaded from the Protein Data Bank (PDB, https://www.rcsb.org/) (ID: 4F6U, resolution: 2.1Å). With the use of ViewerLite v.5, all heteroatoms were removed from the CDK8 structure. Before molecular docking, the energy minimization of the structures was performed using the YASARA Energy Minimization Server (http://www.yasara.org/minimizationserver.htm) [24]. Then, the output SCE files from the YASARA web server were imported into the YASARA View v.20.12.24 software to be saved as PDB files. The binding sites were determined based on a previous study [15]. The molecular docking analysis was carried out using the HADDOCK server (https://wenmr.science.uu.nl/haddock2.4/) [25, 26], and interaction details were analyzed using PyMOL v.2.5.2 and LigPlus + v.2.2.4 [27, 28].

## Results

### Genetic findings

**Fig. 1:**
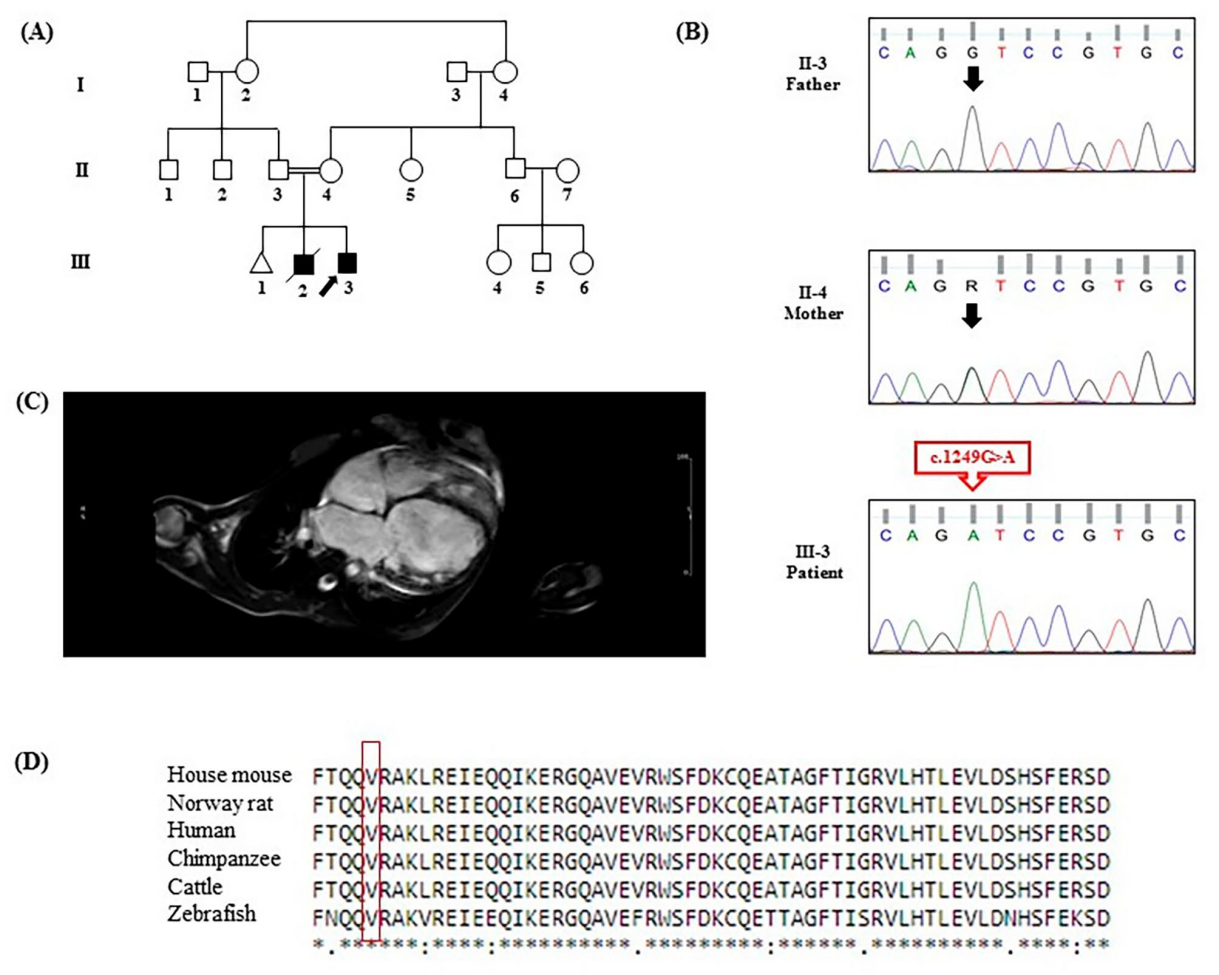
The *MED12* pathogenic variant is responsible for dilated cardiomyopathy (DCM). **(A)** The image presents the pedigree of the family with DCM. Variant carriers: black; relatives without the variant: white; slashed line: the deceased member; square: male; circle: female; arrow: proband; triangle: spontaneous abortion. **(B)** The cardiac magnetic resonance imaging image (CMR) presents dilated cardiomyopathy in the index patient. **(C)** Direct Sanger-sequencing chromatograms show the *MED12* variant sequence in the father, the mother, and the DCM-affected son. The arrow shows the nucleotide position of G/A in the wild-type homozygous father, the heterozygous mother, and the hemizygous patient. **(D)** The CLUSTALW server was used to compare the alignment of MED12 residues among various MED12 orthologs. The valine amino acids are shown in box

WES, performed on the proband (Fig. 1A), identified a novel pathogenic variant, c.1249G > A: p.Val417Ile, in the ninth exon of the *MED12* gene. The c.1249G > A variant was determined as a pathogenic variant (criteria: PM2-Supporting, PP3-Strong, and PP5-Supporting) according to the ACMG guidelines. This variant has not been reported in the 1000 Genomes Project, ExAC, gnomAD, the Human Gene Mutation Database (HGMD), and ClinVar or publications. By using several databases such as CADD (phred = 25.2), SIFT (deleterious = 0.0), PolyPhen-2 (deleterious = 1.0), PROVEAN (deleterious= -2.5), FATHMM (pathogenic= -1.3), and GERP++ (NR = 5), we considered the c.1249G > A missense variant to be the cause of the disease. The candidate variant was confirmed in the proband in a hemizygous state. Other available members of the family, i.e., the healthy mother and the healthy father, were heterozygous state and wild type, respectively (Fig. 1B). The cardiac magnetic resonance imaging image (CMR) finding diagnosed DCM in the proband (Fig. 1C). Furthermore, based on the outcomes from CCLUSTALW, Val417 was identified within the conserved region of the MED12 protein (Fig. 1D).

### Protein-structure modeling and docking

**Fig. 2:**
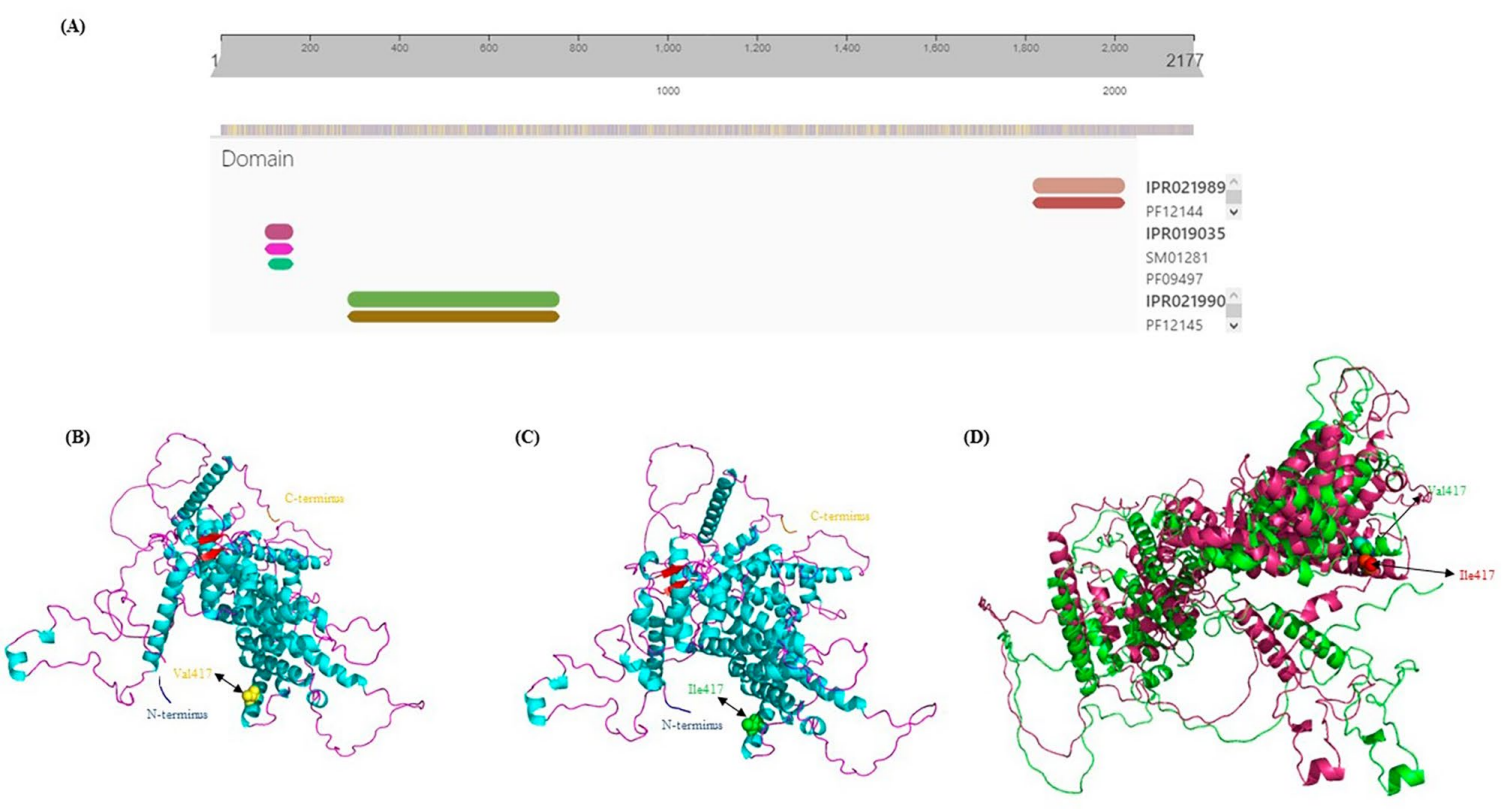
The image depicts the structural prediction of the MED12 protein. **(A)** The image presents the domain prediction, performed with the InterPro web server. **(B)** The computational model of the normal MED12 protein constructed with the aid of the AlphaFold2 web server is presented herein. The surface yellow color shows the normal amino acid. **(C)** The computational model of the variant MED12 protein constructed with the use of the AlphaFold2 web server is presented herein. The surface green color shows the variant amino acid. **(D)** Superimposed AlphaFold2 models of MED12 (amino acid 1–800) as normal (green), Val417Ile (pink)

**Fig. 3:**
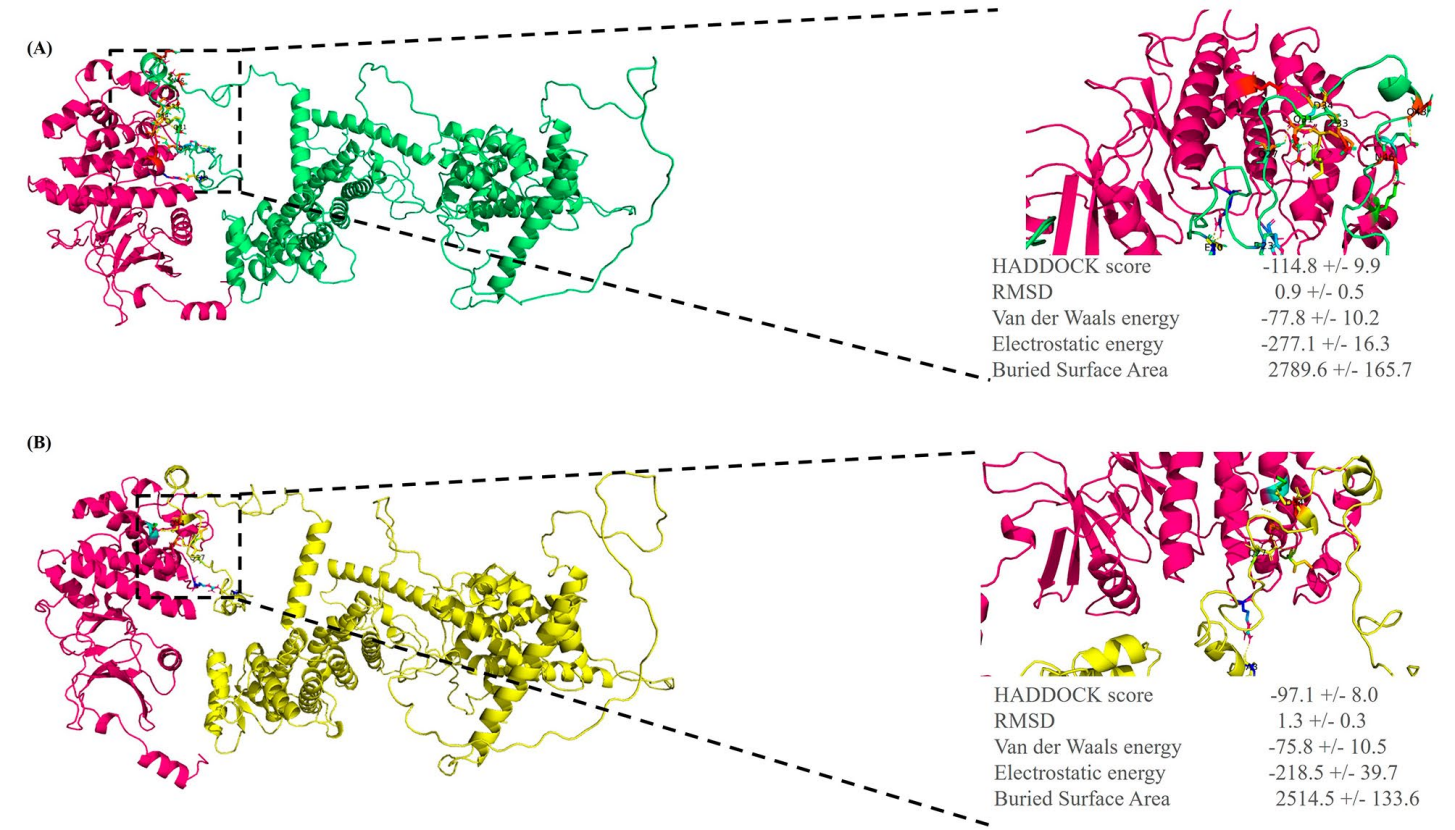
Confidence metrics for the forecasted structure of **(A)** normal MED12 structure and **(B)** Val417Ile variant. The Ramachandran plot illustrates the energetically permissible regions for backbone dihedral angles ψ and ϕ of amino acid residues in the MED12 structure (the normal and the Val417Ile variant). The favored, and allowed regions are depicted in green and blue, respectively. **(C)** The image indicates the possible functions of the native MED12 domains, predicted with the aid of the AlphaFold2 and VAST web servers

The MED12 protein sequence was checked with the InterPro web server, and 3 functional domains were found (Fig. 2A). The amino acids from 1 to 800 that contained the first and second domains of the MED12 protein were used to predict the structure using AlphaFold2. The best models of the human MED12 protein (the normal and the Val417Ile variant) were downloaded from AlphaFold2 using the predicted local distance difference test (pLDDT) scores of 73.3 for both (Fig. 2B and C). pLDDT scores of 0 to 100 indicate the identity of the reproduced model with the reference protein structure. We observed no structural rearrangements in the AlphaFold2 models from MED12 protein (the normal and the Val417Ile variant) (Fig. 2D). The Ramachandran plot was also utilized to assess the structural quality of MED12 protein (the normal and the Val417Ile variant). The percentage of amino acids in the most favored regions, additionally allowed regions, generously allowed regions and disallowed regions of normal MED12, including 86.8%, 10.3%, 0.9% and 2%, and in the Val417Ile variant included 86%, 11.4%, 1.3% and 1.3%, respectively (Fig. 3A and B).

Based on results in the VAST web server, structurally, the sequence containing valine 417 neighbored 266 proteins (Fig. 3C), and Caenorhabditis elegans SYS-1 (PDB ID: 3C2H) had the highest similarity to it. The SYS-1 protein belongs to the β-catenin family [29], which is involved in the transcription and cell-cell adhesion processes. Altered expression profiles (upregulation) of β-catenin have been associated with DCM in humans [30].

**Fig. 4:**
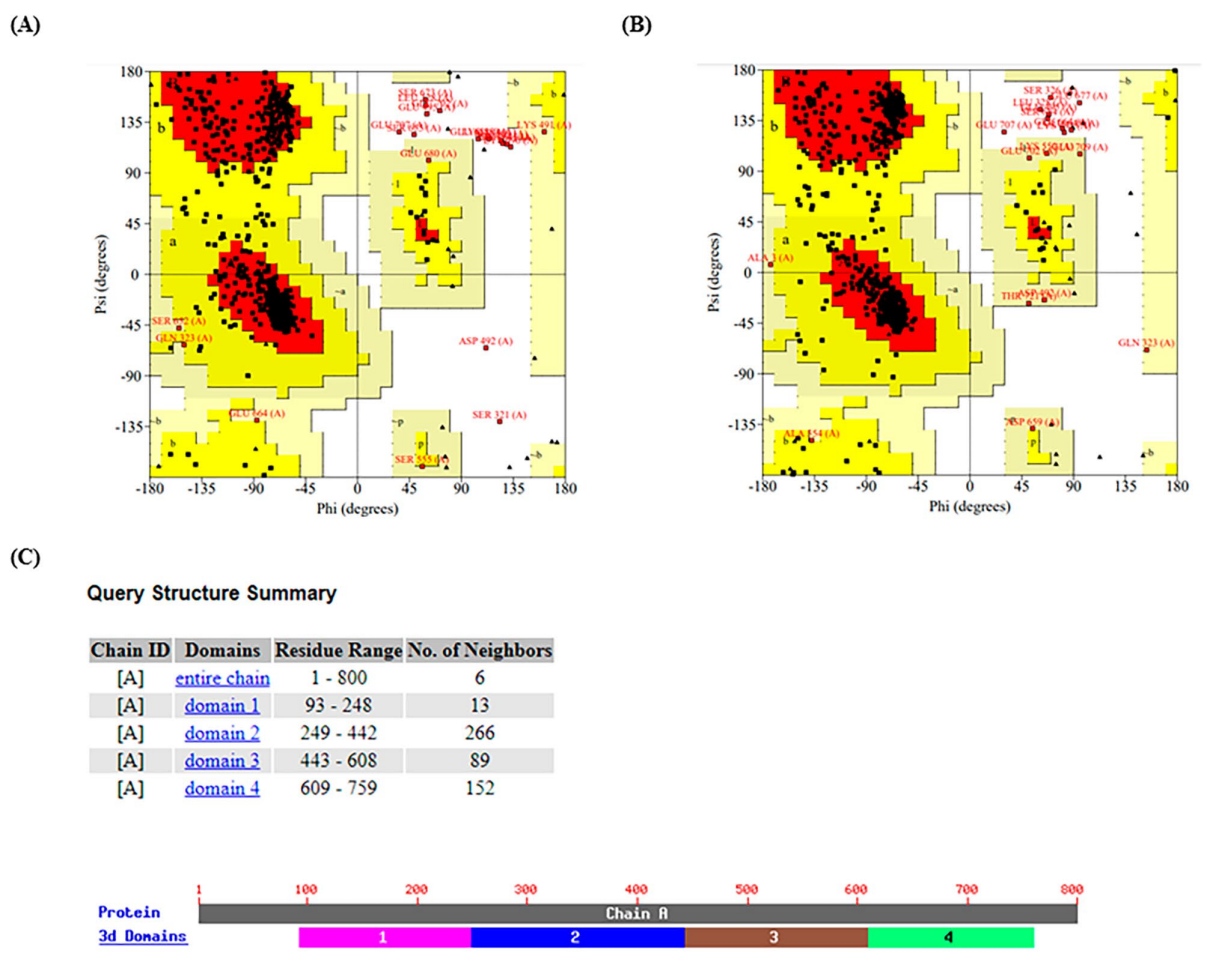
The image demonstrates the molecular docking analysis of the MED12 protein (the normal and variant amino acids) with the CDK8 protein (PDB: 4F6U) by using PyMOL v.2.5.2. **(A)** The image illustrates the protein-protein interactions between the normal MED12 protein and the CDK8 protein (CDK8: purple; normal MED12: green). **(B)** The protein-protein interactions between the MED12 variant and the CDK8 protein (CDK8: purple; variant MED12: yellow) are shown herein

**Fig. 5:**
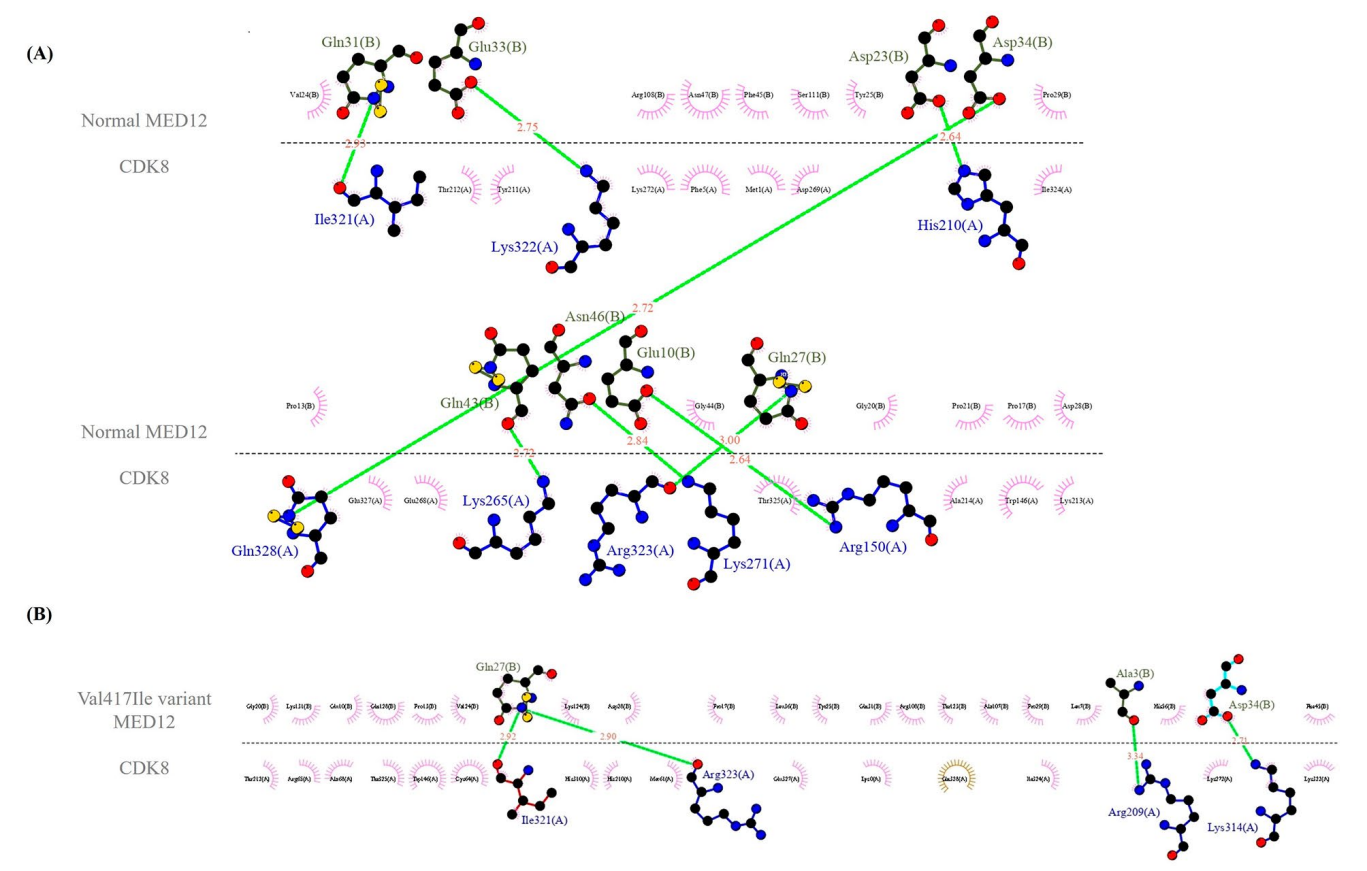
A schematic interaction of the best docking results of the normal **(A)** and variant MED12 **(B)** with CDK8 presented by LigPlus + v.2.2.4 is shown herein. Hydrogen bonding is demonstrated in green

Our modeled MED12 (the normal and the Val417Ile variant) structures and the retrieved CDK8 structure from the PDB database (PDB Id 4F6U) were used for the docking study. A binding site was defined for MED12 (amino acid numbers: 30, 32, 33, 34, 35, 42, 60, 68, 80, 93, 99, 132, 139, and 141) and CDK8 (amino acid numbers: 8, 44, 47, 65, 66, 74, 105, 119, 178, 265, 271, 272, 303, 307, 314, and 322). The docking was completed in the HADDOCK server. The HADDOCK scores of MED12 (the normal and the Val417Ile variant) with CDK8 were − 114.8 ± 9.9 and − 97.1 ± 8.0 with root mean square deviation values of 0.9 ± 0.5 Å and 1.3 ± 0.3 Å, forming 8 and 4 hydrogen bonds, respectively (Fig. 4A and B). Compared with the normal MED12 protein, the Val417Ile variant structure appeared to have a low binding affinity to CDK8. The selected docked cluster of MED12 showed many close interactions with CDK8 and the involved residues, including Gln31, Glu33, Asp23, Asp34, Gln43, Asn46, Glu10, and Gln27, while these residues in the Val417Ile variant model included only Gln27, Ala3, and Asp34 (Figs. 4A and B and 5A, and 5B).

## Discussion

In the present study, we performed WES on a patient with DCM and identified a novel hemizygous variant in *MED12*, c.1249G > A: p.Val417Ile, which was segregated entirely in the family. This novel variant was not found in publications and databases, including the 1000 Genomes Project, ExAC, gnomAD, HGMD, and ClinVar. The Val417Ile variant affected the structure of MED12 in the 3D modeling. Furthermore, our docking analysis revealed an association between this variant and a significant decrease in CDK8 binding, causing a dominant-negative effect on CDK8 activation. Therefore, it seems that the *MED12* variant, c.1249G > A, predisposes the carrier to DCM. The MED12 protein works with numerous transcription factors, including myocyte enhancer factor 2, to regulate the expression of the heart’s calcium-handling genes, such as *ATP2A2*, *GJA1*, *GJA3*, *GJA5*, *KCNN1*, *PLN*, *RYR2*, *TNNT1*, *CACNA1D*, *CASQ1*, and *SLC8A2*, with any expression alteration in these genes causing defects in cardiac function [5]. Indeed, muscle contraction impairment, which occurs in the failing hearts of patients with DCM, is caused by defective calcium (Ca2+) cycling in the sarcoplasmic reticulum due to calcium-handling gene mutations. It is a hallmark of cardiac dysfunction and leads to decreased cardiac contractility and ejection fraction [5, 31]. Any changes in the MED subunits of a mediator complex can be associated with human congenital defects, including congenital heart diseases [32]. The first study to show the involvement of MED in human cardiovascular disorders reported missense mutations in the *MED13L* gene in patients with congenital heart diseases, proving the role of *MED13L* in the early development of the heart [33]. A prior investigation demonstrated that cardiac-specific MED1 deletion, followed by a reduction in calcium-handling gene expression, led to DCM and premature death in mice [34]. MED12 is known to be essential for the early development of mice, especially heart formation, with all mutant *MED12* embryos having an enlarged heart in a previous study [8]. Additionally, Baskin et al. [5] suggested that variants in *MED12* might cause DCM and heart failure. Donnio et al. demonstrated that variations in MED12 are associated with a wide range of genetic disorders related to X-linked intellectual disability, which pose challenges in classification, as they do not align neatly with established syndromes like Lujan, Opitz-Kaveggia, or Ohdo syndromes. In this study, they examined multiple mutations in MED12 among patients (p.R206Q, p.N898D, p.R961W, p.N1007S, p.R1148H, p.S1165P, and p.R1295H). their findings reveal that each MED12 mutation induces distinct expression patterns of immediate early genes (IEGs) such as JUN, FOS, and EGR1 [35]. In the year 2013, three distinct hemizygous missense variants (p.Arg1148His, p.Ser1165Pro, and p.His1729Asn) were identified in three unrelated families afflicted with Ohdo syndrome Maat-Kievit-Brunner (MKB) type. These mutations in MED12 were established as the root cause of this X-linked manifestation of Ohdo syndrome [36].

In conclusion, our results indicate that WES is a feasible approach to discovering new genes and variants in diseases with unknown etiologies. WES can confer the early diagnosis of DCM in affected individuals and their family members at risk for DCM. To our knowledge, the present investigation is the first clinical study to present an association between a *MED12* variant and DCM in humans and, thus, provide new insights into the molecular mechanisms of DCM pathogenesis.

## Data Availability

The datasets generated and/or analyzed during the current study are available in the ClinVar repository [https://www.ncbi.nlm.nih.gov/clinvar/variation/VCV001677246.1/?redir=vcv]. The accession number of the variant in ClinVar is as follows: NM_005120.3 (MED12):c.1249G > A (p.Val417Ile): VCV001677246.1.
